# Influence of EFL Teachers’ Self-Assessment on Their Self-Regulation and Self-Efficacy

**DOI:** 10.3389/fpsyg.2022.891839

**Published:** 2022-04-25

**Authors:** Qian Huang

**Affiliations:** Zhijiang College, Zhejiang University of Technology, Shaoxing, China

**Keywords:** self-assessment, self-efficacy, self-regulation, EFL teachers, teaching performance

## Abstract

This review aimed at investigating the related studies on English as a foreign language (EFL) teachers’ self-assessment and its role in their self-efficacy and self-regulation. Earlier investigations have proved that teacher self-assessment was significantly correlated with self-regulation. Moreover, studies showed that self-assessment and self-regulation enabled teachers to consider their teaching effectiveness, and they were important components of formative assessment. Earlier studies showed that self-assessment raised learner awareness and increased self-efficacy significantly through the improvement of mastery experiences. Furthermore, the study presented the implications and future directions of this line of research for different people, such as EFL teachers, teacher educators, and foreign language scholars. The ideas can improve their awareness of teacher self-assessment, self-regulation, and self-efficacy in educational contexts.

## Introduction

It is supposed that teachers, as the pillars of instructional contexts, help learners to grasp knowledge, attain their full competence, and train learners to have fruitful and constructive life. Those learners are efficacious who are appropriately trained. Consequently, effective teachers are essential for an existing outstanding instructive system. Clotfelter et al., (2007) also believed that learners’ academic success is related to the teachers’ efficiency. Effective teaching is the central component in enhancing the learners’ accomplishments and one of the robust features for the attainment of high-quality learning results. According to Borg and Edmett (2019), teacher self-assessment, as a primary element of teaching effectiveness, is defined as the process of constant reflection, self-checking, and self-decision to uncover the shortcomings which require enhancement. Self-assessment empowers instructors to handle their development. They also asserted that self-assessment is one of the most substantial instruments for the evaluation of teacher quality. Another important element for teaching effectiveness is self-efficacy. Traditionally, investigations have shown that self-efficacy is a significant issue in teaching performance. Therefore, teachers’ attitudes over their capability in language teaching are significant in decision-making practices. Teacher self-regulation is also crucial for teaching evaluation. Capa-Aydin et al. (2009) operationally described teacher self-regulation is “as self-regulatory strategies such as performance monitoring, help-seeking, emotion controlling, and performance/mastery goal setting that EFL teachers implement to attain professional goals” (p. 25). Due to the importance of the above-mentioned variables in teaching effectiveness, this review tries to investigate the role of teachers’ self-assessment in their self-efficacy and self-regulation.

## Literature Review

### Teacher Self-Assessment

According to Pierce (1999), assessment refers to “a beneficial tool which shows teachers and learners that they are making progress in foreign language development, and this would promote their motivation to identify their own strengths and weaknesses and increase their autonomy and independent learning skills” (p. 128). Self-assessment is regarded as one of the numerous assessments that the teachers and learners employ to be aware of their improvement and discern their learning or teaching effectiveness (Asdar, 2017). Goral and Bailey (2019) stated that the tools for learner self-assessment are considered well-organized instruments that contribute to learners realizing the purpose of the task and the appraisal standards. They also mentioned that learner self-assessment is a type of process for accruing and comprehending what learners can or cannot do. Panadero et al. (2017) argued that learners who assess themselves outperform in educational contexts. Numerous investigators considered that self-assessment increases learners’ consciousness to observe their improvement (Meihami and Razmjoo, 2016). It also improves learners’ internal motivation (Yan et al., 2020).

On the other hand, Ross and Bruce (2007) believed that self-assessment is also an appropriate tool for teachers’ professional improvement. Sahli and Benaissi (2019) defined teacher self-assessment as a constant process of the instructors’ attempt to assess teaching efficiency, methodology, abilities, approaches, and viewpoints in order to modify. They also believed that teacher self-assessment expedites learner engagement in academic environments. Bailey (1981) also pointed out that “teacher self-assessment is the process of self-examination in which the teacher uses a series of sequential feedback strategies for the purposes of instructional self-improvement” (p. 9). Compared to numerous surveillances directed by an external assessor, Marzano and Toth (2013) indicated that self-assessments can widely depict a teacher’s capability in instructional contexts. Borg and Edmett (2019) also described teacher self-assessment as a great method for evaluating teacher performance. They also mentioned that teacher self-assessment, compared to other types of teacher evaluation, is the most influential for increasing teaching effectiveness. They stated that teacher self-assessment can be done through self-observing by filling self-evaluation questionnaires following the instruction to assess the pros and cons of instructional performance. Moreover, teachers can record their instruction and activities in class in order to examine their actions meticulously with the help of professionals. Asking learners’ feedback and perceptions about instructional activities can offer prospects for improving the instruction formatively instead of observing the results till the course ends. Moreover, test outcomes and projects are helpful signs of teacher effectiveness and learning achievement (Borg and Edmett, 2019). Therefore, as Allen and Chaerles (2017) pointed out, self-assessment can increase teachers’ affective and cognitive knowledge about their approach to teaching in educational contexts. They also stated that teacher self-assessment increases teacher awareness of learners’ academic requirements.

Some investigations have been done on the role of teacher self-assessment in educational contexts. Lumpe et al. (2000) indicated the significant correlation between teacher self-assessment and motivation in teaching. Moreover, Nova and Sukyadi (2017) also argued that EFL instructors are required to evaluate themselves to build on their relationships with learners. They argued that EFL instructors can foster a positive relationship between themselves and learners through thinking about their own instruction and tasks. Thanh (2019) stated teachers, who use the self-assessment strategy, can do self-reflection to examine the educational context and be accountable for their use of the methodology in EFL contexts. Borgmeier et al. (2016) indicated that teachers’ awareness of practical problems leads to finding solutions to change and ameliorate teaching practice. According to Stronge (2006), “values, beliefs, prior experience, and available school support systems influence the teacher’s perceptions and decisions about both the problem and the possibility of its melioration” (p. 206). In a study on the relationship between teacher self-assessment and teaching experience, Pourjamal Ghouyjagh et al. (2018) found that more experienced EFL teachers’ perceptions of their teaching effectiveness are significantly different from learners’ evaluation of teaching effectiveness. However, they mentioned that working experience helps instructors’ use of conference with other teachers to foster their teaching efficiency.

### The Concept of Teacher Self-Regulation

According to Bandura (1986), self-regulation refers to the “process of setting goals for oneself and engaging in behaviors and cognitive processes that lead to goal completion” (p. 347). Zimmerman (2000) declared that self-regulation is the extent of individuals’ meta-cognitive engagement in educational contexts. He also stated that learners’ degree of motivational and behavioral involvement to reach learning objectives are other indices of self-regulation. According to him, self-regulation is defined as “self-generated thoughts, feelings, and actions that are planned and cyclically adapted to the attainment of personal goals” (p. 14). The self-regulation originates from social cognitive theory of Bandura (1977). Based on this theory, individuals attain their objectives using personal, social, and environmental interaction. Furthermore, self-regulation theory includes self-analysis, self-assessment, and self-response (Bandura, 1977). Schunk and DiBenedetto (2020) argued that individuals, during self-analysis, supervise and record facets of their performance. During self-assessment, they mentioned that individuals detect, assess, and weigh their performance against criteria or objectives. They maintained that individuals in the self-response phase engage their emotions of self-efficacy and emotional responses in their academic performances.

In the instructional contexts, teacher self-regulation indicates instructors’ approaches to control and accomplish their tasks and feelings (Heydarnejad et al., 2017). Mattern and Bauer (2014) stated that teacher self-regulation refers to “strategies that the teachers execute in their work environment to reach professional goals and overcome professional obstacles” (p. 59). There are very few empirical investigations on teacher self-regulation. Toussi et al. (2011) investigated the role of teacher self-regulation in instructional efficiency. They found out that teacher self-regulation, in general, and mastery-goal orientation, emotional control, and intrinsic interest, in particular, significantly predicted the efficiency of instruction. Hamidi and Ghafournia (2021) demonstrated that teachers’ self-regulation is not significantly correlated with their gender. However, they found that instructional experience is significantly correlated with teachers’ self-regulation construct. They argued that self-regulation among teachers is likely to boost with increasing teaching experience. Moreover, they stated that EFL teachers should try to enhance their self-regulation in order to instruct efficiently in educational contexts. In contrast to Hamidi and Ghafournia (2021), Mengistnew et al. (2021) found a significant correlation between gender and teacher self-regulation in that the level of self-regulation is high among females. Moreover, their study revealed a non-significant correlation between job experience and self-regulation among teachers.

Saariaho et al. (2015) reported that teachers are required to have teacher educators’ support to foster their self-regulation through participating in teaching courses for developing their teaching effectiveness. Lee and Turner (2016), in their study, revealed that teachers’ trying to grasp the existing subject matter of tasks to attain the upcoming objectives can significantly predict their self-regulation strategies. They also mentioned that the relationship between teachers’ upcoming purposes and the content of educational courses influences their levels of motivation for teaching and self-regulation. Mattern and Bauer (2014) also showed that instructors’ use of self-regulation strategies improves teaching satisfaction through time-saving and increasing control of workload. They also underscored the appreciated role of teacher education in decreasing job stress through self-regulation strategies.

Considering the correlation between self-efficacy and self-regulation, Ghonsooly and Ghanizadeh (2013) found out that goal setting and mastery goal orientation, as the elements of self-regulation, are significantly correlated with teacher self-efficacy. Furthermore, their study revealed a significant correlation between teachers’ age, years of job experience, and self-regulation. The study of Toussi et al. (2011) also revealed that instructors, who have a great extent of self-regulation trait, are inclined to ascribe their achievement and disappointment in their occupation to self-initiated issues. Their study also showed that self-efficacy cannot moderate the correlation between teachers’ self-initiated change orientation and self-regulation. This implicates that irrespective of self-efficacy, the teacher self-regulation is significantly correlated with his/her self-initiated change. Azari Noughabi and Amirian (2021), in another study, indicated that teacher self-regulation is significantly affected b self-efficacy. They argued that EFL teachers’ use of self-regulatory strategies enhances through the addition of efficacy to their instruction.

### The Notion of Teacher Self-Efficacy

Bandura (1986) defined self-efficacy as “people’s judgments of their capabilities to organize and execute courses of action required to attain designated types of performances” (p. 391). He stated that self-efficacy pinpoints individuals’ self-reliance on their competence to cope with challenging tasks, and to put into practice the prerequisite strategies to be successful in impending situations. Bandura (2011) also stated that individuals’ self-efficacy specifies the way of regulating and managing learning purposes along with persevering in task accomplishment. It also determines the extent of learner resilience and apprehension in coping with difficulties. Investigators have collected considerable evidence underlining the effect of efficacy views, particularly the significant position of teacher self-efficacy on the instruction in educational contexts (Morris et al., 2016). According to Tschannen-Moran et al. (1998), teacher self-efficacy refers to “the teacher’s beliefs in his or her capability to organize and execute courses of action required to successfully accomplish a specific teaching task in a particular context” (p. 233). Tschannen-Moran and Hoy (2001), in another study, considered teacher self-efficacy a dominant issue that verifies failure or achievement in different features of teaching. In order to boost teacher self-efficacy, Bandura (1995) indicated four principal methods, including mastery experiences, vicarious experiences, social persuasion, and physiological and emotional states. Tschannen-Moran and Hoy (2001) stated that the construct of teacher self-efficacy is categorized into classroom management, learner engagement, and teaching strategies. They asserted that classroom management is concerned with instructors’ opinions about their capability to control troublesome behaviors in educational contexts. Moreover, they mentioned that learner engagement denotes teachers’ belief in themselves to be able to involve learners in educational contexts. They also indicated that teaching strategies refer to the instructors’ views about their own capability to be creative in using various appropriate instructional strategies for improving teaching efficiency. Klassen and Tze (2014) asserted that self-efficient teachers outperform in organizing and preparing educational contexts. They are more dedicated to their job to know the requirement of learners. Moreover, they embrace innovations, keep on their profession, and deal with troublesome students (Klassen and Tze, 2014).

Studies have shown that self-efficacy can be regarded as a convincing basis for arousing positive psychology constructs, like wellbeing, and reducing negative emotions like foreign language anxiety. Fathi et al. (2020) found a significant relationship between self-efficacy and psychological wellbeing. They mentioned that job satisfaction and work obligation, along with less anxiety or burnout, are the results of self-efficacy and wellbeing. Based on theory of Bandura (1986), they justified that individuals’ opinions affect both their performance in tasks and thinking patterns and feelings as the critical constructs of psychological wellbeing. Concerning the association between teacher self-efficacy, self-efficacy, and foreign language anxiety, Fathi and Derakhshan (2019) found that well-informed teachers who are able to regulate their academic contexts, reduce foreign language anxiety, employ educational practices, and inspire learners to perform assigned activities are competent in emotional regulation when working on their professional affairs. Ghasemzadeh et al. (2019) found out that teacher self-efficacy, compared to teacher self-reflection significantly, predicts teacher burnout. They argued that teachers who feel less competent in classroom control can feel higher job pressure, which may increase emotional distress and depersonalization. They mentioned that instructors, who suffer depersonalization, no longer show their feelings toward learners. They also justified their study by expressing that the fundamental constituents of teacher self-efficacy are said to predict teacher burnout. They also mentioned that instructors, with high levels of self-efficacy, can innovatively develop and apply various methodologies and teaching techniques, and they can involve learners in order to overcome depersonalization. In line with Ghasemzadeh et al. (2019), Fathi et al. (2021) also revealed that EFL teachers’ self-efficacy, emotional control, and reflection result in the reduction of burnout experiencing in educational contexts and ineffective teaching. They suggested that EFL teachers should participate in preparation agendas to foster their self-efficacy, to reduce stressful situations and alleviate the likelihood of teacher burnout.

### The Role of Teachers’ Self-Assessment in Their Self-Regulation

Self-assessment, as a process of formative assessment, gets individuals to consider the effectiveness of their performance, decide on the extent of meeting the needs and standards, and modify their performance correspondingly (Andrade and Boulay, 2003). Investigations of self-regulation have highlighted the way learners and teachers manage educational processes, whereas studies of self-assessment have emphasized individuals’ decisions of the outcomes of performance (Andrade and Cizek, 2010). Andrade and Cizek (2010) also mentioned that self-assessment and self-regulation engage learners and teachers in reflecting on the effectiveness of their performance, and they are essential components of formative assessment. Zimmerman and Moylan (2009) pointed out that individuals should assess their current reasoning, emotional, emotional, motivational, and interactive processes in order to control and manage themselves. Puustinen and Pulkkinen (2001) also stated that self-assessment is a significant process of regulation theories. However, according to Andrade and Brookhart (2016), “new conceptions of self-assessment are grounded in theories of the self- and co-regulation of learning which includes self-monitoring of learning processes with and without explicit standards” (p. 16). Andrade (2019), in his review, found out that the empirical studies show a significant relationship between self-assessment and self-regulation.

Studies have shown that self-assessment is a precondition for self-regulation and education to happen (Peters and Kitsantas, 2010). Bose and Rengel (2009) designed a model for formative assessment, which could result in the development of self-regulation. They asserted that learners should learn to assess and generate their own work and feedback. Restrepo (2013) empirically studied the effect of self-assessment on learners’ view of their learning. He explored the learners’ viewpoints about learning before and after employing self-assessment. He confirmed that self-assessment could make learners more responsible about their learning, resulting in becoming self-regulated. Furthermore, Sadler (1998) and idea of Brown (2004) in their studies were in favor of using self-assessment because it provides the learners with ample feedback that help the learners self-regulate their learning and set up their own learning goals.

Panadero and Romero (2014), in a study, investigated the effect of the rubric and non-rubric teachers’ self-assessment on their self-regulation. They found that using rubrics in self-assessment improves pre-service teachers’ self-regulation. They argued that the employment of rubrics increases the use of strategies such as planning, monitoring, and evaluation. Panadero et al. (2013) also found out that pre-service instructors who use scripts to assess themselves, in comparison with those who use a rubric for self-assessment, outperform in learning self-regulation. Jafarigohar (2020) found a significant positive role of alternative assessment, like self-assessment, in encouraging the employ of self-regulatory strategies. He argued that self-assessment makes individuals behave autonomously and be accountable for their academic performance in educational contexts. Fathi et al. (2017) also stated that self-assessment in writing composition can enhance EFL individuals’ insight over self-regulation. They argued that providing EFL individuals with the opportunity to evaluate their own writing skill increases the use of self-regulation, including planning and monitoring in writing. Brown and Harris (2014), in their study, revealed that the employment of self-regulation strategies, including the enlightenment of the objectives of the performance, checking the educational procedure and arouse of self-reflection, mediate the significant correlation between self-assessment, and individuals’ educational outcomes. Panadero et al. (2018) stated that self-assessment appears to assist individuals to enhance their self-regulation skills in educational contexts. They mentioned that educational practices must be in accordance with the hypothetical outlines of self-assessment if learners want to acquire self-regulation skills *via* self-assessment. They also argued that self-assessment improves individuals’ performance and empowers them to be accountable for their educational performance.

### The Role of Teachers’ Self-Assessment in Their Self-Efficacy

Some studies have been done on the relationship between learner self-assessment and self-efficacy. Paris and Paris (2001) found a significant effect of self-assessment on learners’ self-efficacy. They argued that learners tend to be successful in their academic performance by being aware of the complexity of the tasks and its requirements. This awareness seems to arouse learners’ confidence in their competence, which ultimately influences self-efficacy. Panadero et al. (2016) stated that helping individuals to reflect on their performance and developing their self-assessment competence provide individuals a feeling of support, agency, and handling their own educational performance, which, ultimately, can boost their self-efficacy. On the other hand, their study revealed that reflection and self-assessment after revision significantly influence learner self-efficacy and they improve writing skill. Yan et al. (2020) found out that self-efficacy did not mediate the correlation between self-assessment and academic achievement. Their study also revealed that self-assessment significantly improves self-efficacy through the enhancement of mastery experiences. Kissling and O’Donnell (2015) also argued that self-assessment raises learner awareness and increase self-efficacy. They mentioned that when learners pay attention to numerous dimensions of their speech, they promote the mastery experiences through which self-efficacy increases. Baleghizadeh and Masoun (2014) also approved that self-assessment in educational contexts enhances self-efficacy and self-regulation.

Yan (2020) argued that, following self-assessment, learners may put more effort into learning through asking for more feedback on their performance which increases learner self-efficacy. In another study on the effect of feedback on self-assessment and self-efficacy, Kim and Lee (2019) found that instructors’ oral feedback significantly affects the accurateness of self-assessment along with feelings and self-efficacy. They suggested that teachers should consider the type of positive or negative feedback since their study revealed that negative feedback offered the opportunity for learners to be careful in self-assessment. However, this type of provided feedback reduces learner self-efficacy.

Andrade et al. (2009) investigated the effect of a rubric for self-assessment on learners’ self-efficacy. They found out that learners’ self-efficacy increased the usage of the rubric for self-assessment. Panadero et al. (2017), in their study, revealed that gender mediates the correlation between self-assessment and self-efficacy. Their study indicated that females’ self-efficacy, compared to boys’, fosters more utilizing self-assessment interventions. Nieminen et al. (2021), in their study, suggested that shifting the model of self-assessment, from formative to summative one, can increase the level of self-efficacy. Chung et al. (2021) investigated the effect of self-assessment in post- and pre-revision conditions on learners’ writing performance and self-efficacy. They found out that involving learners in reflection, planning, and goal determination as to the processes of self-assessment before revision is effective for increasing self-efficacy. The studies which investigate the relationship between teacher self-assessment and teacher self-efficacy are meager. Mahasneh and Alwan (2018) demonstrated that project-based learning as a kind of digital assessment significantly builds pre-service teachers’ self-efficacy. They mentioned that teachers in, project-based learning, were required to develop and bring to an end the projects in which they face extensive difficulties to which they are required to explore answers. They argued that problem solving takes the time of pre-service teachers in the process of completing project-based learning which increases teachers’ mastery experience.

## Implications and Suggestions for Further Research

This review inspected the related literature on the effect of teachers’ self-assessment on their self-regulation and self-efficacy. It improves the educational knowledge of investigators who are interested in learner self-assessment, self-regulation, and self-efficacy. Regarding the related literature about the positive role of self-efficacy and self-regulation in educational and instructional performance, it is worth noting that learners should be helped to regulate their emotions in educational environments. Learners can improve their efficacy by selecting easy-to-difficult authentic tools based on their language proficiency level. Teachers can provide promising educational context for learners to arouse their efficacy. Teachers can enhance learner self-regulation by providing positive feedback and particular hints for employing self-regulatory strategies. They can model reflective practices or engender groups for discussion through collaborative learning in order to practice reflection on issues. They can also employ experiential learning tasks and incorporate real-life tasks into educational contexts. If the teachers use formative assessment and prominently the associated strategies, the learners will learn how to assess their progress, regulate their learning, and employ effective methods and strategies to achieve the best result. All of these result in becoming self-regulated learners. Moreover, in formative assessment learners are in the center of teaching and learning. They learn collaboratively with the others in class. Consequently, they are trained to self-assess their progress based on the assessments and become eager to use their teachers’ feedback, which results in enhancing autonomy. Therefore, the teachers can consider this point and use it in their classes to improve learners’ autonomy. Moreover, it is suggested to use self-assessment, and its related strategies in English language classrooms to motivate and enhance learners’ critical thinking.

Teachers can also promote learner self-efficacy by providing an environment for learners to observe peers’ achievement in a task. This induces learners’ self-confidence since they can perform their tasks self-reliantly. Teachers can boost learners’ self-efficacy through constructive rapport. They can also provide helpful comments to encourage learners to engage in educational contexts. Also, knowledge of learners’ personality types may inspire teachers to employ teaching principles to enhance learner self-efficacy in educational contexts. Therefore, L2 teachers are needed to interact with learners about their internal and external motivation and ask their problems to improve their positive viewpoints over educational contexts. Also, paying attention to needs analysis should be one of the first principles of teaching to achieve educational outcomes. Therefore, they need to modify the materials based on learner competence to build learner self-efficacy. Instructors can use moderately difficult activities, which can empower learners with low levels of self-efficacy. The activities should not be too difficult to curb learner self-confidence in doing tasks. Teacher support, including scaffolding, assigning sufficient time, decomposing difficult tasks into simple phases, and explicating the task, is influential for the enhancement of learner self-efficacy. This can produce an insight of reasonable challenge and equalizes the complexity of tasks.

Praising and giving feedback to learners are also important for the improvement of learner self-efficacy. Moreover, teachers should not compare the performances of learners with each other. Teachers can provide learners with some strategies such as self-verbalization. For example, they can motivate learners to express the procedure of learning grammatical points or vocabulary aloud and give feedback on their effort. Moreover, teachers can set a cooperative context, rather than a competitive one, to increase learner interaction and scaffolding, which can improve learner self-efficacy. They can also ask learners to write comments about their feelings and progressions in educational contexts. They may also change their methodology by taking self-efficacy into account in their instruction. They can offer warming-up activities for educational contexts and brainstorm learners to increase self-efficacy in educational contexts. The projects, lectures, conferences, and workshops may put extra stress on learners and decrease their self-efficacy. In order to increase learner self-efficacy, familiarizing learners with questions of the tests can be helpful. Providing a competitive educational context through quizzes augments learner self-efficacy. Unplanned quizzes are predominantly important for stimulating learners with less self-efficacy levels. Teachers can provide learners with some video files like TED videos in online classrooms, and they can discuss the effect of self-efficacy on the improvement of individuals’ performance.

Teachers can also improve learners’ self-assessment by providing a context for summative assessment by asking them to score their own academic performance. They can also provide contexts for constant organized formative learning by the employment of online tests that provide learners instant feedback on their performance. After a test, learners commonly concentrate on their grades instead of focusing on the way their used strategies and approaches affect their grades. Teachers can use exam wrappers, as a new instrument, to assist learners in highlighting three points associated with the exam: (1) their planning before the test, (2) their weak points in the test; and (3) the alternations that they can make prior to the upcoming test. Moreover, having learners create a reflective journal about their education and attainments is a reasonable method to involve learners in assessing themselves and it can offer some perceptions about learners’ proficiency to teachers. Teachers can integrate self-assessment into any assessment activities. Using technologies, including journals or blogs, to handle self-assessment can also encourage learners to engage in self-assessment.

This study has some implications for teacher educators, policymakers, and advisors. In order to improve self-efficacy and self-regulation, teacher educators and mentors can provide a situation in which teachers can observe the instruction of their peers. Teacher educators can also emphasize instructors to attach importance to the constant academic development and critical thinking to enhance their instructional method. Instructors should be directed to be well informed about instructive issues and take advantage of improved learning chances. It is also suggested that teacher educators highlight interaction tools, like mobile applications, which encourage teachers and learners to interact and scaffold that increase self-efficacy and self-regulation. They should develop confidence and competence among in-service teachers to entice learners’ interests and engage them in the learning process. Educational policymakers should hire experienced teachers, as the instructive experience can be an important issue for increasing self-efficacy among teachers. Educational policymakers increase learner self-efficacy by holding academic workshops that offer learners and teachers some authentic activities. They can ask learners and teachers to do their best within varied educational contexts. Therefore, the validity of experience, the arrangement of the contexts, and the observation of the learners can lead to the reduction of task difficulty and improvement in learners’ skills. The importance of self-efficacy and self-regulation can motivate consultants to expand their horizons to identify learners’ and teachers’ sources of efficacy and self-regulation to remove their obstacles.

In future work, investigating learner self-assessment and its role in learner self-regulation and self-efficacy in technology-supported contexts, numerous cultural backgrounds, and among learners with different language proficiency levels can be important for future studies. Some investigations need to be done on the effect of teacher self-assessment on learner motivation in traditional and virtual contexts. Furthermore, the relationship between teacher proficiency level of foreign language and its effect on their self-assessment in educational contexts should be considered for the future. Furthermore, case and phenomenological investigations, which provide us the reasons behind teacher self-efficacy and self-regulation, are required to be done. Some studies should be done on the relationship between positive psychological constructs such as enjoyment, grit, positive affectivity, resilience, and self-assessment. In addition, future research should examine the relationship between other negative factors such as boredom, burnout, anger, frustration, and self-assessment. Further research is needed to inspect the relationship between learner motivation, teacher self-efficacy, and self-regulation. Moreover, the effect of the other formative assessment strategies can be investigated on different skills such as speaking and writing. In addition, some studies can be conducted on the self-assessment of different ranges of age, including adults and children.

## Author Contributions

The author confirms being the sole contributor of this work and has approved it for publication.

## Conflict of Interest

The author declares that the research was conducted in the absence of any commercial or financial relationships that could be construed as a potential conflict of interest.

## Publisher’s Note

All claims expressed in this article are solely those of the authors and do not necessarily represent those of their affiliated organizations, or those of the publisher, the editors and the reviewers. Any product that may be evaluated in this article, or claim that may be made by its manufacturer, is not guaranteed or endorsed by the publisher.

## References

[ref1] Allen J. Chaerles E. (2017). Teacher Self-Assessment: A Facilitative Process for Principal Instructional Support. doctoral dissertation. Committee Faculty of the College of Education: University of Houston.

[ref2] Andrade H. L. (2019). A critical review of research on student self-assessment. Front. Educ. 4:87. doi: 10.3389/feduc.2019.00087

[ref3] Andrade H. G. Boulay B. A. (2003). Role of rubric-referenced self-assessment in learning to write. J. Educ. Res. 97, 21–30. doi: 10.1080/00220670309596625

[ref4] Andrade H. Brookhart S. M. (2016). “The role of classroom assessment in supporting self-regulated learning,” in Assessment for Learning: Meeting the Challenge of Implementation. eds. Laveault D. Allal L. (Cham: Springer), 293–309.

[ref5] Andrade H. Cizek G. J. (eds.) (2010). “Students as the definitive source of formative assessment: academic self-assessment and the self-regulation of learning,” in Handbook of Formative Assessment. New York: Routledge, 102–117.

[ref6] Andrade H. Wang X. L. Du Y. Akawi R. L. (2009). Rubric-referenced self-assessment and self-efficacy for writing. J. Educ. Res. 102, 287–302. doi: 10.3200/JOER.102.4.287-302

[ref7] Asdar A. (2017). Students’ self–assessment on their spoken interaction using CEFR. Proc. Educ. Lang. Int. Conf. 1, 148–161.

[ref8] Azari Noughabi M. Amirian S. M. R. (2021). Assessing the contribution of autonomy and self-efficacy to EFL teachers’ self-regulation. Eng.Teach. Learn. 45, 71–88. doi: 10.1007/s42321-020-00060-4

[ref9] Bailey G. D. (1981). Teacher Self-Assessment: A means for Improving Classroom Instruction. New York: National Education Association.

[ref10] Baleghizadeh S. Masoun A. (2014). The effect of self-assessment on EFL learners’ self-efficacy. TESL Canada J. 31, 42–58. doi: 10.18806/tesl.v31i1.1166

[ref11] Bandura A. (1977). Social Learning Theory. Oxford, England: Prentice-Hall.

[ref12] Bandura A. (1986). Social Foundations of Thought and Action: A Social Cognitive Theory. Englewood Cliffs, NJ: Prentice Hall.

[ref13] Bandura A. (ed.) (1995). Self-Efficacy in Changing Societies. Cambridge: Cambridge University Press.

[ref14] Bandura A. (2011). A social cognitive perspective on positive psychology. Revista De Psicologia Soc. 26, 7–20. doi: 10.1174/021347411794078444

[ref15] Borg S. Edmett A. (2019). Developing a self-assessment tool for English language teachers. Lang. Teach. Res. 23, 655–679. doi: 10.1177/1362168817752543

[ref16] Borgmeier C. Loman S. L. Hara M. (2016). Teacher self-assessment of evidence-based classroom practices: preliminary findings across primary, intermediate and secondary level teachers. Teach. Dev. 20, 40–56. doi: 10.1080/13664530.2015.1105863

[ref17] Bose J. Rengel Z. (2009). A model formative assessment strategy to promote student-centered self-regulated learning in higher education. Online Subm. 6, 29–35.

[ref18] Brown D. H. (2004). Language Assessment: Principles and Classroom Practices. New York, NY: Pearson Education, Inc.

[ref19] Brown G. T. Harris L. R. (2014). The future of self-assessment in classroom practice: reframing self-assessment as a core competency. Frontline Learn. Res. 2, 22–30. doi: 10.14786/flr.v2i1.24

[ref20] Capa-Aydin Y. Sungur S. Uzuntiryaki E. (2009). Teacher self-regulation: examining a multidimensional construct. Educ. Psychol. 29, 345–356. doi: 10.1080/01443410902927825

[ref21] Chung H. Q. Chen V. Olson C. B. (2021). The impact of self-assessment, planning and goal setting, and reflection before and after revision on student self-efficacy and writing performance. Read. Writ. 34, 1885–1913. doi: 10.1007/s11145-021-10186-x

[ref22] Clotfelter C. Ladd H. Vigdor J. (2007). Teacher credentials and student achievement in high school: a cross-subject analysis with student fixed effects. J. Hum. Resour. 45, 655–681. doi: 10.1353/jhr.2010.0023

[ref23] Fathi J. Derakhshan A. (2019). Teacher self-efficacy and emotional regulation as predictors of teaching stress: an investigation of Iranian English language teachers. Teach. Eng. Lang. 13, 117–143. doi: 10.22132/TEL.2019.95883

[ref24] Fathi J. Derakhshan A. Saharkhiz Arabani A. (2020). Investigating a structural model of self-efficacy, collective efficacy, and psychological well-being among Iranian EFL teachers. Iran. J. App. Lang. Studies 12, 61–80. doi: 10.22111/ijals.2020.5725

[ref25] Fathi J. Greenier V. Derakhshan A. (2021). Teacher self-efficacy, reflection, and burnout among Iranian EFL teachers: the mediating role of emotion regulation. Iran. J. Lang. Teach. Res. 9, 13–37.

[ref26] Fathi J. Mohammad Yousefi L. Sedighravesh M. (2017). The impact of self-assessment and peer-assessment in writing on the self-regulated learning of Iranian EFL students. J. Sociol. Res. 8, 1–16. doi: 10.5296/jsr.v8i2.11252

[ref27] Ghasemzadeh S. Nemati M. Fathi J. (2019). Teacher self-efficacy and reflection as predictors of teacher burnout: an investigation of Iranian English language teachers. Issues Lang. Teach. 8, 25–50. doi: 10.22054/ilt.2020.49009.451

[ref28] Ghonsooly B. Ghanizadeh A. (2013). Self-efficacy and self-regulation and their relationship: a study of Iranian EFL teachers. Lang. Learn. J. 41, 68–84. doi: 10.1080/09571736.2011.625096

[ref29] Goral D. P. Bailey A. L. (2019). Student self-assessment of oral explanations: use of language learning progressions. Lang. Test. 36, 391–417. doi: 10.1177/0265532219826330

[ref30] Hamidi S. Ghafournia N. (2021). Investigating the relationship among Iranian EFL teachers’ self-regulation, effective teaching, gender, and teaching experience. Int. J. Foreign Lang. Teach. Res. 9, 167–186.

[ref31] Heydarnejad T. Hosseini Fatemi A. Ghonsooly B. (2017). Emotions and self-regulation: a case of Iranian EFL high school and private language institute teachers. Int. J. Educ. Invest. 4, 82–100.

[ref32] Jafarigohar M. (2020). The effect of assessment technique on EFL learners’ writing motivation and self-regulation. Iran. J. Engl. Acad. Purp. 9, 141–162.

[ref33] Kim E. J. Lee K. R. (2019). Effects of an examiner’s positive and negative feedback on self-assessment of skill performance, emotional response, and self-efficacy in Korea: a quasi-experimental study. BMC Med. Educ. 19, 1–7. doi: 10.1186/s12909-019-1595-x, PMID: 31088436 PMC6518717

[ref34] Kissling E. M. O’Donnell M. E. (2015). Increasing language awareness and self-efficacy of FL students using self-assessment and the ACTFL proficiency guidelines. Lang. Aware. 24, 283–302. doi: 10.1080/09658416.2015.1099659

[ref35] Klassen R. M. Tze V. M. C. (2014). Teachers’ self-efficacy, personality, and teaching effectiveness: a meta-analysis. Educ. Res. Rev. 12, 59–76. doi: 10.1016/j.edurev.2014.06.001

[ref36] Lee J. Turner J. (2016). The role of pre-service teachers’ perceived instrumentality, goal commitment, and motivation in their self-regulation strategies for learning in teacher education courses. Asia Pac. J. Teach. Educ. 45, 213–228. doi: 10.1080/1359866X.2016.1210082

[ref37] Lumpe A. T. Haney J. J. Czerniak C. M. (2000). Assessing teachers’ beliefs about their science teaching context. J. Res. Sci. Teach. 37, 275–292.

[ref38] Mahasneh A. M. Alwan A. F. (2018). The effect of project-based learning on student teacher self-efficacy and achievement. Int. J. Instr. 11, 511–524. doi: 10.12973/iji.2018.11335a

[ref39] Marzano R. J. Toth M. (2013). Teacher Evaluation that Makes a Difference: A New Model for Teacher Growth and Student Achievement. Alexandria, VI: ASCD.

[ref40] Mattern J. Bauer J. (2014). Does teachers’ cognitive self-regulation increase their occupational wellbeing? The structure and role of self-regulation in the teaching context. Teach. Teach. Educ. 43, 58–68. doi: 10.1016/j.tate.2014.05.004

[ref41] Meihami H. Razmjoo S. A. (2016). An emic perspective toward challenges and solutions of self-and peer-assessment in writing courses. Asian-Pac. J. Sec. Foreign Lang. Educ. 1, 1–20. doi: 10.1186/s40862-016-0014-7

[ref42] Mengistnew M. Sahile A. Asrat D. (2021). Examining teachers’ self-regulation practice in secondary school science teaching: the case of South Gondar zone. Ethiopia. Heliyon 7, 1–9. doi: 10.1016/j.heliyon.2021.e08306, PMID: 34820533 PMC8601992

[ref43] Morris D. B. Usher E. L. Chen J. A. (2016). Reconceptulizing the sources of teaching self-efficacy: a critical review of emerging literature. Educ. Psychol. Rev. 29, 795–833. doi: 10.1007/s10648-016-9378-y

[ref44] Nieminen J. H. Asikainen H. Rämö J. (2021). Promoting deep approach to learning and self-efficacy by changing the purpose of self-assessment: a comparison of summative and formative models. Stud. High. Educ. 46, 1296–1311. doi: 10.1080/03075079.2019.1688282

[ref45] Nova M. Sukyadi D. (2017). An innovation of teacher’s self-assessment of rapport building in EFL classroom. Proc. Educ. Lang. Int. Conf. 1, 496–507.

[ref46] Panadero E. Alonso-Tapia J. Reche E. (2013). Rubrics vs. self-assessment scripts effect on self-regulation, performance and self-efficacy in pre-service teachers. Stud. Educ. Eval. 39, 125–132. doi: 10.1016/j.stueduc.2013.04.001

[ref47] Panadero E. Andrade H. Brookhart S. (2018). Fusing self-regulated learning and formative assessment: a roadmap of where we are, how we got here, and where we are going. Aust. Educ. Res. 45, 13–31. doi: 10.1007/s13384-018-0258-y

[ref48] Panadero E. Brown G. T. Strijbos J. W. (2016). The future of student self-assessment: a review of known unknowns and potential directions. Educ. Psychol. Rev. 28, 803–830. doi: 10.1007/s10648-015-9350-2

[ref49] Panadero E. Jonsson A. Botella J. (2017). Effects of self-assessment on self-regulated learning and self-efficacy: four meta-analyses. Educ. Res. Rev. 22, 74–98. doi: 10.1016/j.edurev.2017.08.004

[ref50] Panadero E. Romero M. (2014). To rubric or not to rubric? The effects of self-assessment on self-regulation, performance and self-efficacy. Assess. Educ. Principles, Policy, Prac 21, 133–148. doi: 10.1080/0969594X.2013.877872

[ref51] Paris S. G. Paris A. H. (2001). Classroom applications of research on self-regulated learning. Educ. Psychol. 36, 89–101. doi: 10.1207/S15326985EP3602_4

[ref52] Peters E. E. Kitsantas A. (2010). The effect of nature of science metacognitive prompts on science students’ content and nature of science knowledge, metacognition, and self-regulatory efficacy. Sch. Sci. Math. 110, 382–396. doi: 10.1111/j.1949-8594.2010.00050.x

[ref53] Pierce L. V. (1999). Preparing independent learners: The role of self-assessment. Revista Canaria de Estudios Ingleses 38, 127–137.

[ref54] Pourjamal Ghouyjagh H. Sotoudehnama E. Faghih E. (2018). On the impact of teaching experience on EFL instructors’ self-assessment of their instructional effectiveness. Iran. J. Engl. Acad.Purp 6, 47–70.

[ref55] Puustinen M. Pulkkinen L. (2001). Models of self-regulated learning: a review. Scand. J. Educ. Res. 45, 269–286. doi: 10.1080/00313830120074206

[ref56] Restrepo A. (2013). Role of systematic formative assessment on students’ views of their learning. Prof. Issues Teachers Prof. Develop 15, 165–183.

[ref57] Ross J. A. Bruce C. D. (2007). Teacher self-assessment: a mechanism for facilitating professional growth. Teach. Teach. Educ. 23, 146–159. doi: 10.1016/j.tate.2006.04.035

[ref58] Saariaho E. Pyhältö K. Toom A. Pietarinen J. Soini T. (2015). Student teachers’ self- and co-regulation of learning during teacher education. Learn. Res. Prac 2, 44–63. doi: 10.1080/23735082.2015.1081395

[ref59] Sadler D. R. (1998). Formative assessment: revisiting the territory. Assess. Educ 5, 77–84. doi: 10.1080/0969595980050104

[ref60] Sahli N. Benaissi F. B. (2019). Using teacher self-assessment and reflection to foster change in the writing classroom. Int. J. Soc. Sci. Educ. Stud 5:39. doi: 10.23918/ijsses.v5i4p39

[ref61] Schunk D. H. DiBenedetto M. K. (2020). Motivation and social cognitive theory. Contemp. Educ. Psychol. 60, 101832–101847. doi: 10.1016/j.cedpsych.2019.101832

[ref62] Stronge J. H. (2006). “Teacher evaluation and school improvement,” in Evaluating Teaching: A Guide to Current Thinking and Best Practice. ed. Stronge H. J. (Thousand Oaks, CA: Corwin), 1–22.

[ref63] Thanh N. T. (2019). Promoting learner autonomy through self-assessment and reflection. VNU J. Foreign Stud 35, 146–153. doi: 10.25073/2525-2445/vnufs.4483

[ref64] Toussi M. T. M. Boori A. A. Ghanizadeh A. (2011). The role of EFL teachers’ self-regulation in effective teaching. World J. Educ. 1, 39–48. doi: 10.5430/wje.v1n2p39

[ref65] Tschannen-Moran M. Hoy A. W. (2001). Teacher efficacy: capturing an elusive construct. Teach. Teach. Educ. 17, 783–805. doi: 10.1016/S0742-051X(01)00036-1

[ref66] Tschannen-Moran M. Hoy A. W. Hoy W. K. (1998). Teacher efficacy: its meaning and measure. Rev. Educ. Res. 68, 202–248. doi: 10.2307/1170754

[ref67] Yan Z. (2020). Self-assessment in the process of self-regulated learning and its relationship with academic achievement. Assess. Eval. High. Educ. 45, 224–238. doi: 10.1080/02602938.2019.1629390

[ref68] Yan Z. Chiu M. M. Ko P. Y. (2020). Effects of self-assessment diaries on academic achievement, self-regulation, and motivation. Assess. Educ. Prin. Policy Prac 27, 562–583. doi: 10.1080/0969594X.2020.1827221

[ref69] Zimmerman B. J. (2000). “Attaining self-regulation: a social cognitive perspective,” in Handbook of Self-Regulation. eds. Boekaerts M. Pintrich P. R. Zeidner M. (San Diego, CA: Academic Press), 13–39.

[ref70] Zimmerman B. J. Moylan A. R. (2009). “Self-regulation: where metacognition and motivation intersect,” in Handbook of Metacognition in Education. eds. Hacker D. J. Dunlosky J. Graesser A. C. (New York: Routledge), 299–315.

